# POWTEX visits POWGEN

**DOI:** 10.1107/S1600576723002819

**Published:** 2023-04-25

**Authors:** Andreas Houben, Yannick Meinerzhagen, Noah Nachtigall, Philipp Jacobs, Richard Dronskowski

**Affiliations:** a Chair of Solid-State and Quantum Chemistry, Institute of Inorganic Chemistry, RWTH Aachen University, D-52056 Aachen, Nordrhein-Westfalen, Germany; b Chair of Glass and Glass-Ceramic, Institute of Mineral Engineering, RWTH Aachen University, D-52074 Aachen, Nordrhein-Westfalen, Germany; Tohoku University, Japan

**Keywords:** neutron detectors, POWGEN beamline, POWTEX detector, DREAM beamline, time-of-flight diffraction, angular-dispersive refinement, wavelength-dispersive refinement, powder diffraction, Rietveld refinement, multi-dimensional refinement

## Abstract

The high-intensity time-of-flight (TOF) neutron diffractometer POWTEX for powder and texture analysis is currently being built prior to operation in the eastern guide hall of the research reactor FRM II at Garching close to Munich, Germany. Because of the world-wide ^3^He crisis in 2009, the authors promptly initiated the development of ^3^He-free detector alternatives that are tailor-made for the requirements of large-area diffractometers. Herein is reported the 2017 enterprise to operate one mounting unit of the final POWTEX detector on the neutron powder diffractometer POWGEN at the Spallation Neutron Source located at Oak Ridge National Laboratory, USA. As a result, presented here are the first angular- and wavelength-dependent data from the POWTEX detector, unfortunately damaged by a 50*g* shock but still operating, as well as the efforts made both to characterize the transport damage and to successfully recalibrate the voxel positions in order to yield nonetheless reliable measurements. Also described is the current data reduction process using the *PowderReduceP2D* algorithm implemented in *Mantid* [Arnold *et al.* (2014). *Nucl. Instrum. Methods Phys. Res. A*, **764**, 156–166]. The final part of the data treatment chain, namely a novel multi-dimensional refinement using a modified version of the *GSAS-II* software suite [Toby & Von Dreele (2013). *J. Appl. Cryst.* **46**, 544–549], is compared with a standard data treatment of the same event data conventionally reduced as TOF diffraction patterns and refined with the unmodified version of *GSAS-II*. This involves both determining the instrumental resolution parameters using POWGEN’s powdered diamond standard sample and the refinement of a friendly-user sample, BaZn(NCN)_2_. Although each structural parameter on its own looks similar upon comparing the conventional (1D) and multi-dimensional (2D) treatments, also in terms of precision, a closer view shows small but possibly significant differences. For example, the somewhat suspicious proximity of the *a* and *b* lattice parameters of BaZn(NCN)_2_ crystallizing in *Pbca* as resulting from the 1D refinement (0.008 Å) is five times less pronounced in the 2D refinement (0.038 Å). Similar features are found when comparing bond lengths and bond angles, *e.g.* the two N—C—N units are less differently bent in the 1D results (173 and 175°) than in the 2D results (167 and 173°). The results are of importance not only for POWTEX but also for other neutron TOF diffractometers with large-area detectors, like POWGEN at the SNS or the future DREAM beamline at the European Spallation Source.

## Abstract 2

The first real-world neutron diffraction data have been collected with one of the POWTEX detectors (FRM II, Garching, Germany) mounted for testing at the Spallation Neutron Source (Oak Ridge National Laboratory, USA). They allow for angular- and wavelength-dispersive Rietveld refinement using a modified version of *GSAS-II*.

## 1. Introduction

The future POWTEX instrument is a pulsed high-intensity time-of-flight (TOF) diffractometer at a continuous reactor source. Its design utilizes several novel concepts and is suited to small sample quantities (Conrad *et al.*, 2008 ▸; Houben *et al.*, 2012 ▸). The jalousie-design large-area detector system is tailor-made to fulfill the resolution requirements and instrument characteristics of POWTEX and has been described in detail already (Modzel *et al.*, 2014 ▸). The almost blind-spot free alignment of the cylindrical shape around the spherical sample is realized by two detector types following the same detection principle. The major instrument components of POWTEX, including the surface detectors at 2θ ≃ 45–135°, have been manufactured. Because of past and present delays not further commented on here, we cannot yet operate POWTEX at the Forschungsreaktor München (FRM) II, despite having actively developed multi-dimensional data treatment and analytical methods for angular- and wavelength-dependent diffraction data (Jacobs *et al.*, 2015 ▸, 2017 ▸). There has therefore been an obvious need to find an appropriate place to test our brand new 240 cm long detectors under realistic (*i.e.* POWTEX-like) diffraction conditions. The POWGEN instrument at the Spallation Neutron Source (SNS), Oak Ridge National Laboratory (ORNL), USA, was an ideal choice for several reasons. First, POWGEN is a latest-generation TOF diffractometer with a huge large-area detector coverage (Huq *et al.*, 2011 ▸). Second, prior to an instrument upgrade program, there was sufficient space to install one POWTEX detector mounting unit centered at 2θ = 90° at the future POWTEX sample-to-detector distance of 80 cm. Third, we knew the POWGEN instrument quite well from user beam times and from our methodological work (Jacobs *et al.*, 2013 ▸), for which comparing the two instrument designs seems very promising.

Testing the POWTEX detector was also important as regards its younger sister, DREAM, to be operated at the European Spallation Source (ESS), Lund, Sweden, since the same jalousie-type detector concept has been chosen for the DREAM diffractometer with only minor modifications. Because a major international project such as the ESS is intensely monitored and thoroughly analyzed in all aspects, POWTEX’s detector concept, the associated methods developed and the projected test must also have appeared convincing in this dense field of competition, because they also indirectly touch upon DREAM.

It took one year from presenting our ideas to Ashfia Huq, the leading POWGEN instrument scientist at that time, until the scheduled experiment in November 2017, carefully and narrowly timed between the end of the user program cycle and POWGEN’s own upgrade program thereafter, which would permanently occupy the space used to test the POWTEX detector. After many documentations, agreements and further meetings, the detector was shipped to ORNL, where it finally arrived with several 50*g* shock-watches having been triggered and the detector being most probably damaged. In a way, this very shock translated into the instrument developers, too. Because of the unique chance to take measurements with the POWTEX detectors at POWGEN, we tried to ship a replace­ment detector but it would not have arrived on time. After preliminary investigation of the damage, several anode wires turned out to be broken, at least one corner of the detector hood was dented, and a slight rattling sound could be heard from within when the detector was shaken gently. This being a fine piece of precision German engineering, we still took the opportunity to use the test beam time in order to characterize the damage and possibly learn about the detector as well.

## 2. Beam time and raw data conversion

**Figure 1:**
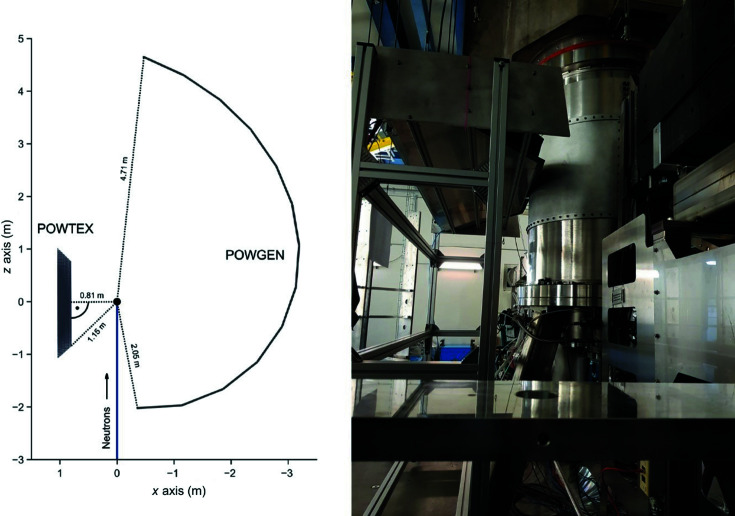
(Left) A schematic drawing showing one of the POWTEX cylinder surface detector mounting units (left-hand part) mounted as a pack of eight detector modules in the POWTEX detector geometry. It is aligned orthogonal to 2θ = 90° in the horizontal scattering plane (paper plane) at a distance of 81 cm (instead of the ideal 80 cm) from the sample at the center of the coordinate system. The maximum angular coverage of this type of POWTEX detector is Δ2θ = 90°. Each mounting unit covers 9° in the φ direction (almost perpendicular to the paper plane). The drawing results from the instrument definition file (IDF) as used with *Mantid*. The much larger POWGEN detector system is shown on the opposite hemisphere. (Right) A photograph of the detector (plus mockup and shielding) touching the POWGEN detector vessel, *i.e.* the neutron window, as closely as possible to give the best match to the ideal 80 cm sample-to-detector distance.

In contrast to earlier tests targeting detector physical properties (Modzel *et al.*, 2014 ▸), this beam time aimed to collect diffraction data with a setup as close as possible to the later POWTEX setup (see Fig. 1 ▸). While the entire primary instrument (neutron guides, choppers, slits *etc.*) was different from POWTEX, *i.e.* identical to the POWGEN instrument, the secondary instrument consisted completely of POWTEX components (except for the neutron windows in the POWGEN sample vessel). This means that, in addition to the detector itself, the analog and digital electronics, the clock synchronization distribution, the high-voltage supply, the gas-handling system, the detector firmware *etc.* were also all used as planned for POWTEX. The *T*_0_ chopper signal of POWGEN was fed into our detector clock to synchronize with the SNS pulse generation.

All diffraction patterns discussed in the following were measured with POWGEN’s high-resolution chopper setting at 60 Hz in ‘Bank 1’, *i.e.* using a center wavelength of λ_c_ = 0.7 Å and, accordingly, a wavelength range of λ = 0.2–1.2 Å; the angular coverage is Δ2θ ≃ 90°. Note that during data reduction the raw data coverage in 2θ and λ is typically reduced by masking/cutting away ambiguous ranges (as an example, see Section 4 and Fig. 6).

In the main test phase, the setup was run continuously for six days without any failure, constantly collecting neutron events. As a sad consequence of the transport damage, two and a half of the eight subunits had to be disabled completely because their anode wires were broken, producing a short circuit. It may well be the case that the remaining five and a half subunits are the only 50*g* shock-proven neutron detectors world wide.

In addition to the standard samples used to calibrate the POWGEN instrument, *e.g.* highly crystalline diamond powder, a vanadium rod, an NAC sample (Na_2_Ca_3_Al_2_F_14_, space group *I*2_1_3, *a* = 10.251 Å) and empty POWGEN sample holders (background), three real-world user beam times by ‘friendly scientists’ were simultaneously measured on the POWGEN instrument and on the POWTEX detector. Herein, we will only refer to the data sets for diamond, vanadium, background and BaZn(NCN)_2_. Vanadium primarily scatters incoherently and isotropically in space, allowing for a correction of the detector efficiency per detector voxel, *i.e.* a pixel of a volume detector. Furthermore, geometric issues such as the Lorentz effect (Kisi & Howard, 2008 ▸; Jacobs *et al.*, 2015 ▸) and the source spectrum, *i.e.* the TOF-dependent intensity distribution on the path from the source to the sample, are intrinsically accounted for. The background measurement is subtracted to remove ‘noise’ from other scattering sources. The diamond sample with its very sharp reflections is used to correct the detector voxel positions (or TOF offsets) within the overall instrument geometry and compared with the ideal instrument definition.

Since the POWTEX detector was running continuously, the continuous raw data files were first cut into subsets according to the time stamps of the NeXus (Könnecke *et al.*, 2015 ▸) files generated for POWGEN using a routine supplied by Günter Kemmerling (JCNS, Jülich, Germany) to yield a set of data files correlated with the simultaneous measurements using the POWGEN detector. Afterwards, those raw files were converted to NeXus files by a conversion routine supplied by Gerd Modzel (CDT GmbH, Germany, later at JCNS). During the conversion, the metadata (sample name, chopper, instrument settings *etc.*) which were identical for both experiments were copied from the POWGEN NeXus file, while the event data were converted from the raw POWTEX data collected at a given time stamp. All further analysis and data treatment routines described below have been designed to work with the NeXus format, since it will be the future event file format for POWTEX at FRM II.

For a future machine such as POWTEX, a neutron event in a TOF experiment using a volume detector consists of three spatial coordinates and a time channel. However, in NeXus files only voxel identifiers as written by the firmware are stored, while the mapping to (diffraction) coordinates needs to be done separately and as needed for the data treatment. In *Mantid* (Arnold *et al.*, 2014 ▸) this is achieved by an instrument definition file (IDF), which was created according to the analysis of the transport damage. Simply speaking, it holds each voxel’s center of gravity position and its hexahedron-like shape connected with a unique detector identifier (ID). In the design of the POWTEX jalousie detector, each voxel has a slightly different (individual) shape.

## 3. Analysis of transport damage

The installation and alignment of each instrument component involve small deviations from the ideal positioning as defined in the IDF, especially in a test setup, and even more so if the detector has been damaged. Therefore, there are deviations (Δ*d*) between the theoretical and actual voxel positions of the detector. Consequently, discrepancies exist regarding the interplanar distances *d* that are of final interest and calculated from these deviating voxel positions. Δ*d* is determined routinely on neutron diffractometers, *e.g.* at the beginning of each measurement cycle using a standard sample. For POWGEN, this is usually a polycrystalline powdered diamond sample. For technical reasons, the differences are treated by the introduction of a correction factor dif*C*′ by analogy with the conventional dif*C* [see equation (1)] relating the measured time of flight (TOF_exp_) and its corresponding *d* value,

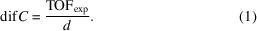

All possible effects causing the spatial dislocation of a voxel (alignment effects, detector damage *etc.*) need to be corrected by this factor prior to further evaluation of the usual diffraction data sets.

**Figure 2:**
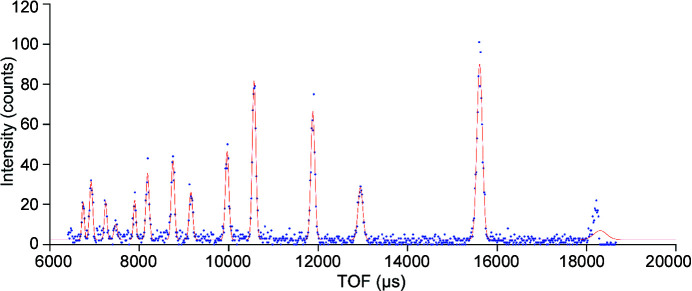
An example diffractogram of the TOF values measured similarly for each voxel. The experimental data points are shown as blue dots and the fitted function as a red line.

The first step in correcting the voxel positions is to determine the current TOF value for each peak on each voxel. The NeXus files contain the TOF values for each neutron event in a voxel with its respective detector ID. The diffractograms for each voxel (Fig. 2 ▸) are fitted with a constant peak profile function. Each peak is defined by the theoretical *d* value, by the peak shape parameters and, most importantly, by the offset value to be determined. With the exception of this offset, the other peak shape parameters should be known in advance, as the offset value must be fitted to the experimental data based on a known peak shape and position.

It is of crucial significance that the description of the experimental data be consistent throughout the entire process of data treatment. For example, it is quite common to use either pseudo-Voigt functions (pV) or pV functions convoluted with back-to-back exponentials (Von Dreele *et al.*, 1982 ▸) (pV–b2b) for the correction procedure. Since the SNS is a short-pulse source, the reflections show the known asymmetries, and it is advisable to use the back-to-back exponentials for the correction in this case, as is done for the POWGEN instrument (Ikeda & Carpenter, 1985 ▸; *FULLPROF* manual, https://www.ill.eu/sites/fullprof/; Rodríguez-Carvajal, 1993 ▸). Unfortunately, for cases of many voxels and reflections this is computationally demanding, especially when using the back-to-back exponentials. Herein, only the Gaussian part (Gaussian–b2b) was used for the correction (equivalent to a pV–b2b mixing parameter η = 0). Since this choice has no effect on the *d* offset it is a safe step to do. For additional time saving, only the Gaussian functions were used in an initial run to calculate the starting values of *S* and *H* [see equations (2)–(6) [Disp-formula fd4] [Disp-formula fd5] [Disp-formula fd6]] for a second fit using pV–b2b functions.

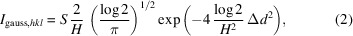




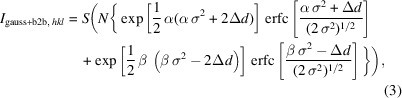




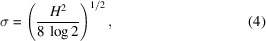




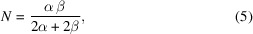




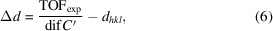

In these equations, *S* = scale, *H* = FWHM, Δ*d* = offset in ångströms, *t* = TOF, dif*C*′ = corrected dif*C* in µs Å^−1^, α, β = rise and decay constants, respectively, and *d_hkl_* = theoretical *d* value of the peak. The fit results in a data set with dif*C*′ values for each detector ID (voxel). From the offset and the theoretical peak positions, an experimental TOF value can be calculated via 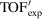 = *d*_*hkl*_ dif*C*′.

With 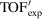 , *d_hkl_*, and the uncorrected *x*, *y* and *z* coordinates of each voxel, new *x*′, *y*′ and *z*′ coordinates can be calculated:

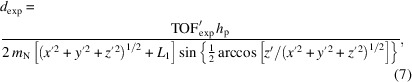




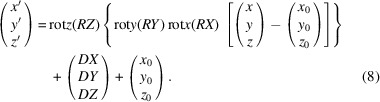

Equation (7) is deduced from Bragg’s law, de Broglie’s equation and the instrument geometry. *L*_1_ is the primary flight path from source to sample (60 m), (*x*_0_, *y*_0_, *z*_0_) is the center of the detector, rot*x*, rot*y* and rot*z* are the rotation matrices around the individual coordinate axes with the respective rotation angles (*RX*, *RY*, *RZ*), (*DX*, *DY*, *DZ*) is the translation vector, *h*_p_ is the Planck constant, and *m*_N_ is the neutron mass. The rotation and translation parameters *RX*, *RY*, *RZ* and *DX*, *DY*, *DZ* given in equation (8), being the same for the entire mounting unit, are fitted to yield alternative voxel coordinates until the experimental *d*_exp_ positions for all reflections match the theoretical *d_hkl_* positions.

Using this set of parameters, the global detector geometry could be determined, and a new IDF was created giving all corrected voxel coordinates.

**Figure 3:**
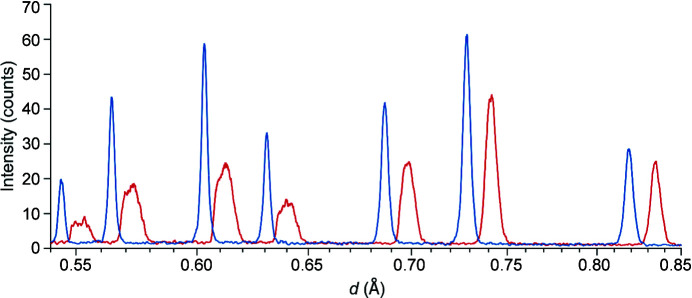
A one-dimensional diffractogram for the diamond sample measured on POWTEX at POWGEN, in red for the uncorrected and in blue for the corrected detector alignment accounting for the detector damage.

Fig. 3 ▸ shows the difference between the uncorrected (red) and corrected (blue) detector alignment in a one-dimensional diffractogram (*I* versus *d*). Two effects can be observed: there is a general shift in the *d* position of the peaks and an apparent broadening of the peak shape.

**Figure 4:**
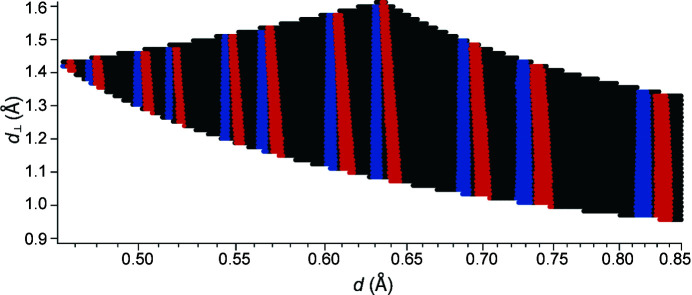
A two-dimensional diffractogram for the diamond POWTEX at POWGEN data sets, with an uncorrected (red) and corrected (blue) detector alignment also handling the effects of the detector damage. By the definition of 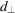 , the corrected reflections (blue) need to be exactly vertical for each *d*_*hkl*_ value. The widths of the blue and red reflections in this plot are proportional to the FWHM for each reflection and are meant just as a guide to the eye.

The deviations regarding the peak shapes can be better illustrated in a two-dimensional drawing of a new kind. Fig. 4 ▸ shows the same data set as in Fig. 3 ▸ as a two-dimensional 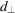 –*d* scheme reduced to provide only information about the peak positions and thus neglecting intensities. To introduce the 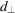 variable with respect to the ordinate, one expresses *d* through λ and θ by the Bragg equation while 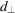 becomes

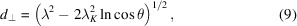

such that 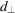 (= λ at θ = 0; 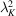 = 1 Å^2^) is an alternative coordinate which – together with *d* – gives a new orthogonal coordinate system (Jacobs *et al.*, 2015 ▸, 2017 ▸). Fig. 4 ▸ reveals that the peaks do not necessarily broaden, as assumed from the 1D view in Fig. 3 ▸, but that the *d* position is shifted to smaller *d* values over the course of 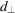 . As a result, the red peaks in Fig. 4 ▸ are skewed and, accordingly, the red reflections in Fig. 3 ▸ are broadened and deformed. Upon inclusion of the applied correction (blue), the peaks are aligned along 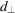 at constant *d* values, as required by the definition, and also show a correct one-dimensional diffractogram (Fig. 3 ▸). Having thus acquired both ingredients, namely NeXus files like those for the future POWTEX diffractometer and a corrected IDF describing the detector alignment and the detector damage, it is then possible to investigate the *Mantid* routines for handling those data sets.

## 4. Data reduction in *Mantid*

**Figure 5:**
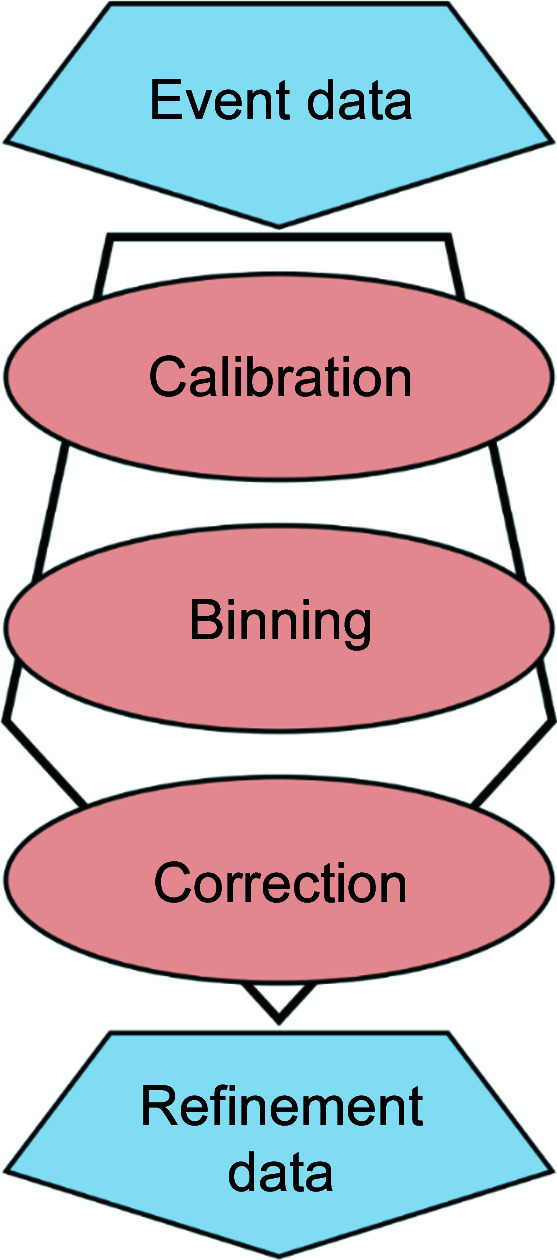
The three basic steps of data reduction applied by the workflow algorithm *PowderReduceP2D* to convert raw event data to refineable .p2d data.

Reduction of the raw event data has been accomplished using the *Mantid* software (Arnold *et al.*, 2014 ▸; https://www.mantidproject.org/). As there was no existing algorithm for reducing angular- and wavelength-dependent data, the new workflow algorithm *PowderReduceP2D* was implemented into the *Mantid* software. *PowderReduceP2D* (see the *Mantid* documentation for technical details; https://docs.mantidproject.org/nightly/algorithms/PowderReduceP2D-v1.html) reduces raw event data following three basic steps (see Fig. 5 ▸), calibration, binning and correction of the raw event data.

For the reduction of raw sample data, two standard measurements should be supplied as well, namely measurements of an empty sample holder and a vanadium sample. The empty measurement serves to reduce background noise, while the vanadium measurement allows the user to normalize differences in detector voxel efficiency and other effects (Jacobs *et al.*, 2017 ▸). During the reduction, all three sets of raw data are basically treated the same. Only the vanadium set needs some additional corrections which will be explained in the relevant steps below.

**Figure 6:**
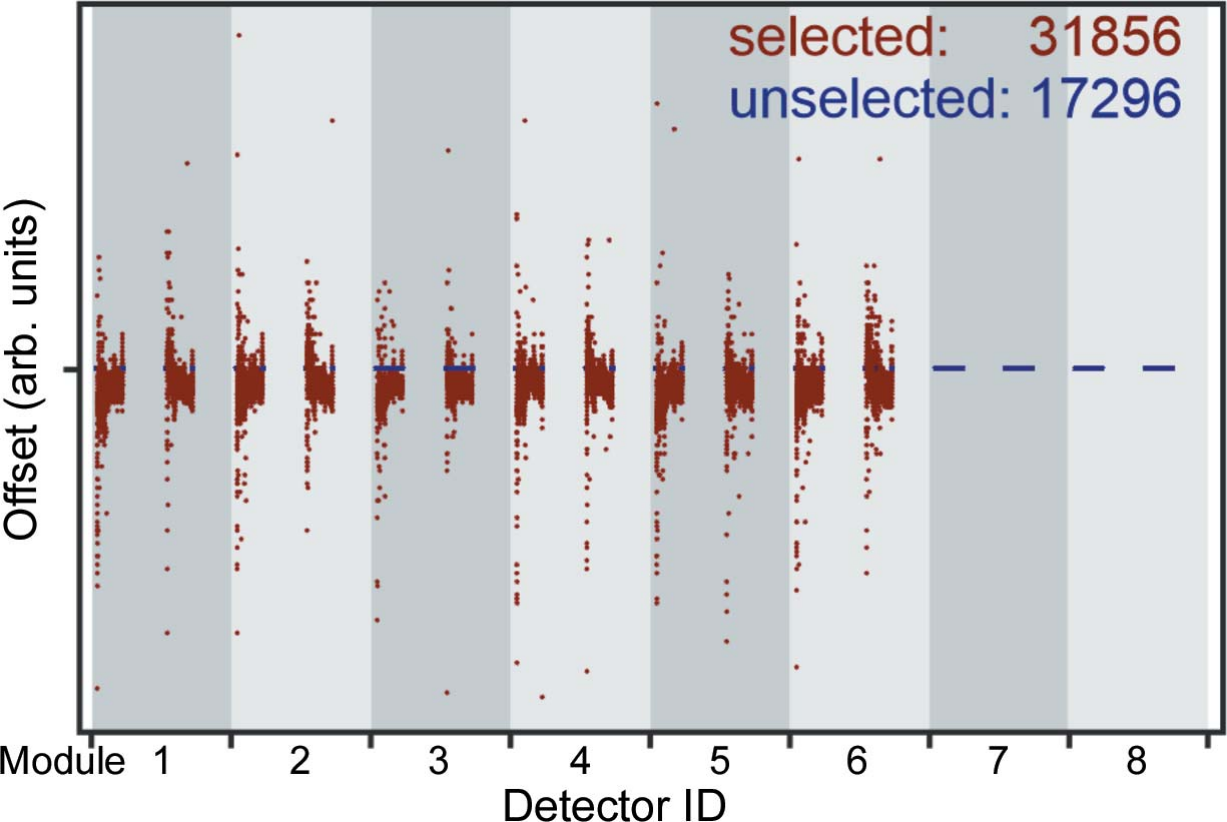
A diagram showing the offsets assigned to each voxel as identified by their detector ID. Red voxels were selected for further data treatment while blue voxels, all having an offset of exactly zero, were masked for different reasons. Each of the eight detector modules per mounting unit consists of two subunits. Therefore, the four subunits of modules 7 and 8 were masked because their anode wires were broken. Similarly, in the third module, half of the detector volume (in depth) had to be shut down.

The first step, calibration, applies several already existing *Mantid* algorithms for inspecting the neutron pulse, calibrating the detector positions, and masking the detectors if needed. The algorithm *FilterBadPulses* is used to remove events that occurred while the proton charge was below a certain threshold. Additionally, the algorithm *RemovePromptPulse* (as one can guess from its name) removes the prompt pulse from the measured data. The detector geometry is computed using the algorithm *FindDetectorsPar* and further calibrated by the algorithm *AlignDetectors. AlignDetectors* uses a calibration file that was once generated using the routine *CalibrateRectangularDetectors* (used until *Mantid* Version 6.2), with the diamond measurement and a list of reflection positions as input, again to correct detector positions. In contrast to the calibration procedure described above, this function is only capable of handling tiny displacements of individual voxels. As described in the section above, it was necessary first to adjust the voxel positions in the IDF in order to describe the alignment and detector damage effects for the detector as a whole, and afterwards to realign the individual voxel positions to generate the required calibration files for the subsequent routines. Certain voxels showing overly large offsets or malfunctioning were also masked, using the algorithm *MaskDetectors* (see Fig. 6 ▸).

In the case of reducing raw vanadium event data, further processing is done at this point. The algorithm *CylinderAbsorption* is applied to calculate correction factors for attenuation caused by absorption and scattering inside a cylindrical sample.

The second step for data reduction is the binning of the raw event data in two dimensions. The algorithm *Bin2DPowderDiffraction* either bins the event data in classical linear/logarithmic bins or applies the recently developed edge binning (Jacobs, 2017 ▸). If edge binning is used, a file containing bin limits has to be supplied. In the case of linear/logarithmic binning, it is sufficient to supply a bin width (conventions as also used for other routines: positive bin width for linear binning, negative bin width for logarithmic binning). In the case of binned vanadium data, further processing using the algorithm *StripVanadiumPeaks* is necessary to remove the coherent intensity contributions (reflections). The algorithm *FFTSmooth* is then used to compensate noise, utilizing the Fourier transform to filter higher frequencies.

The third step for data reduction is the correction of the measured data. The correction follows equation (10) for each bin,

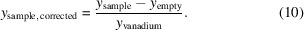

**Figure 7:**
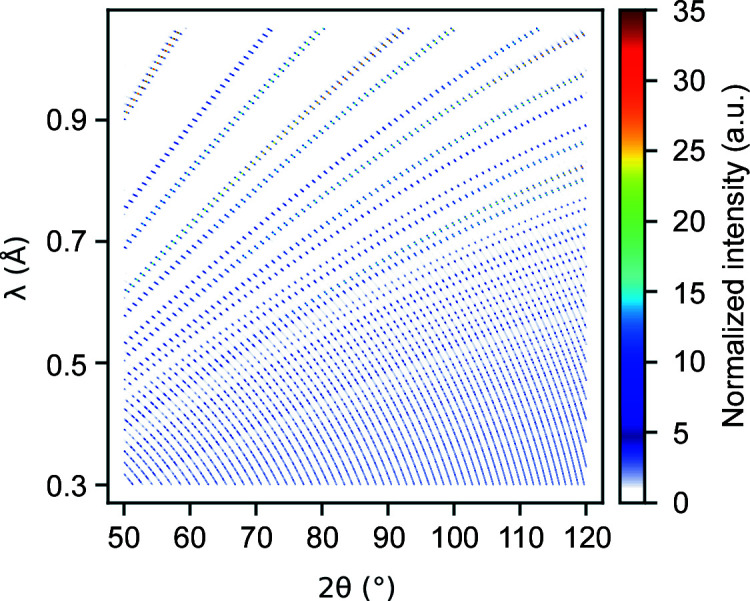
Angle- and wavelength-dispersive diffraction pattern (2θ–λ plot) of the reduced observed data for the diamond sample

First, the empty measurement data are bin-wise subtracted from the sample measurement data to remove any background noise created by the sample holder. Second, the resulting data are divided by the vanadium measurement data to compensate for any differences in detector voxel efficiencies *etc*. Finally, a text file containing the fully reduced two-dimensional sample data is created with the suffix .p2d by calling the *SaveP2D* algorithm of *Mantid*. A .p2d file contains information about the instrument used and the applied binning in the header, and data columns for 2θ, λ, *d*, *d*_⊥_, the binned intensity and its standard deviation. Additionally, the function allows the user to select the data range to write into the .p2d file, in both 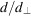 and 2θ/λ space; Fig. 7 ▸ provides a plot of such data in 2θ/λ coordinates. This data file can then be used as input data for refinement software like *GSAS-II* (Toby & Von Dreele, 2013 ▸), which was modified to perform a structure refinement with multi-dimensional data according to the Rietveld method (see Section 5). Additionally, an instrument parameter file is required for the refinement process.

Next to the instrument setup, a set of instrument-specific parameters is given, for example for the analytical calculation of the peak width and shape during the refinement. These parameters are usually determined according to a well known setup by measuring a reference sample, in this case diamond, and they remain unchanged in subsequent refinements of user data. Herein, an extended parametrization comparable to the one described by Jacobs *et al.* (2017 ▸) for the POWGEN instrument was used. The details of this parametrization clearly go beyond the scope of this article and will be reported separately in the future.

## 5. Multi-dimensional refinement

For refining the created .p2d data files, *GSAS-II* was modified to treat multi-dimensional neutron TOF data. In this study two example refinements of diamond and BaZn(NCN)_2_ are shown.

The refinement of the diamond data [for structural information see Straumanis & Aka (1951 ▸)] was accomplished in four steps:

(i) Background refinement using a two-dimensional Chebyshev formula (Chebyshev, 1858 ▸) with 15 parameters in each dimension. The procedure is very similar to the conventional treatment.

(ii) Refinement of cell parameters.

(iii) Refinement of isotropic displacement parameters.

(iv) Refinement of instrumental parameters for further use, since diamond is a standard sample.

For comparison, a conventional 1D refinement was also carried out using *GSAS-II*. To do so, the raw data were reduced with the standard data reduction algorithm used at POWGEN, *i.e.* the algorithm *SNSPowderReduction* in *Mantid*; the same POWTEX IDF was used as that developed herein. In the refinement, the usual pV–b2b profile function as for POWGEN was used to determine the instrumental parameters using the diamond data set, analogous to the procedure described above for two dimensions in steps (i)–(iv). Note that in particular the b2b parameters α and β cannot be refined but need to be tuned manually. This is the same for the conventional POWGEN procedure. As explained, the resulting instrumental resolution parameters were also kept constant for the refinement of the structural parameters of the second sample, as shown in the next section.

For the described conventional refinements, the unmodified *GSAS-II* code yielded identical results to the *GSAS-II* revision including our modifications.

**Figure 8:**
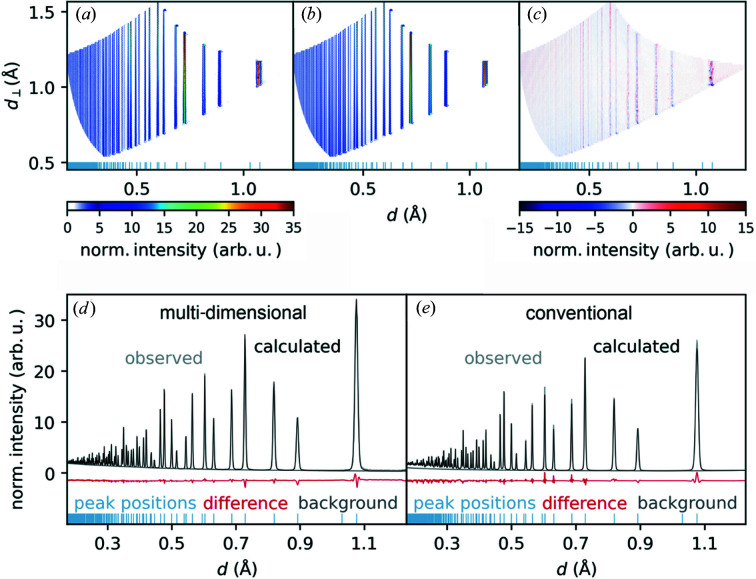
Results of the multi-dimensional Rietveld refinement for a neutron TOF measurement of diamond. (*a*) The observed pattern, (*b*) the calculated pattern, (*c*) the difference pattern, (*d*) a 1D plot of multi-dimensional refinement and (*e*) the conventional one-dimensional refinement. Blue lines indicate peak positions. The color scale shows normalized intensity in plots (*a*)–(*c*).

The result of the multi-dimensional refinement is depicted in Fig. 8 ▸, in particular showing in Fig. 8 ▸(*a*) the observed pattern, in Fig. 8 ▸(*b*) the calculated pattern, in Fig. 8 ▸(*c*) the difference pattern and in Fig. 8 ▸(*d*) a one-dimensional ‘standard’ plot of the multi-dimensional refinement. For comparison, Fig. 8 ▸(*e*) depicts a conventional one-dimensional refinement of the same measured data but conventionally reduced. The color scale shows normalized intensity values because the sample measurement was divided by a vanadium measurement during data reduction. It is immediately obvious that the calculated pattern is a particularly good representation of the measured data according to the structural description of the diamond sample. There are only minor deviations visible in Fig. 8 ▸(*c*), located at the peak positions and correlating with the peak intensity. The corresponding *R*_Bragg_ values and cell parameters are given in Table 1 ▸. While the only carbon atom position on the special Wyckoff site 8*a* is fixed, the isotropic displacement parameters arrived at *U*_iso_ = 0.0005 (1) Å^2^ in the conventional and *U*_iso_ = 0.00171 (1) Å^2^ in the multi-dimensional refinement.

To demonstrate that the multi-dimensional Rietveld refinement can not only be easily applied to highly crystalline diamond samples (for which also all correction work was done), a data set from a real-world (and current) user sample, BaZn(NCN)_2_, was analyzed as a second example. This phase is a ternary carbo­diimide (a nitro­gen-based pseudo-oxide with a complex NCN^2−^ anion) with tetrahedrally coordinated Zn^2+^ and eightfold Ba^2+^ coordination, crystallizing in *Pbca* with *a* = 11.934, *b* = 11.927 and *c* = 6.845 Å from powder X-ray diffraction (XRD) (Corkett *et al.*, 2018 ▸). Here, the data range in *d* was restricted to 0.65–1.08 Å due to challenges in calculating a multi-dimensional background description. The refinement of the BaZn(NCN)_2_ data was accomplished in four steps:

(i) Background refinement using a two-dimensional Chebyshev formula (Chebyshev, 1858 ▸) with 15 parameters in each dimension.

(ii) Refinement of cell parameters.

(iii) Refinement of isotropic dis­place­ment parameters.

(iv) Refinement of atomic positions.

Instead of refining the instrument parameters, we re-used the results from the diamond refinement.

**Figure 9:**
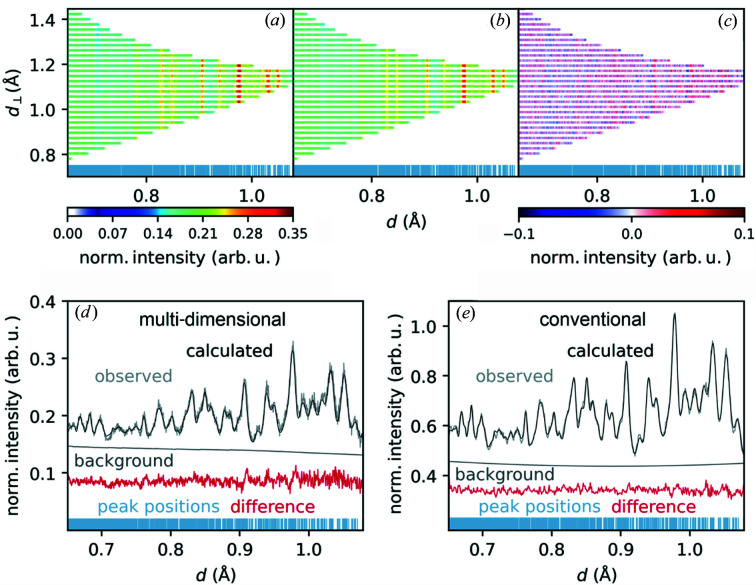
Results of the multi-dimensional Rietveld refinement for a neutron TOF measurement of BaZn(NCN)_2_. (*a*) The observed pattern, (*b*) the calculated pattern, (*c*) the difference pattern, (*d*) a 1D plot of multi-dimensional refinement and (*e*) the conventional one-dimensional refinement result. Blue lines indicate peak positions. The color scale shows normalized intensity in plots (*a*)–(*c*).

The result of the refinement is displayed in Fig. 9 ▸, showing in Fig. 9 ▸(*a*) the observed pattern, in Fig. 9 ▸(*b*) the calculated pattern, in Fig. 9 ▸(*c*) the difference pattern and in Fig. 9 ▸(*d*) a one-dimensional ‘standard’ plot of the multi-dimensional refinement. Again, Fig. 9 ▸(*e*) offers a conventional one-dimensional refinement of the likewise conventionally reduced measured data for comparison. The color scale refers to normalized intensity because the sample measurement was renormalized by division of the vanadium measurement. Figs. 9 ▸(*a*) and Fig. 9 ▸(*b*) differ strongly from the corresponding plots in Fig. 8 ▸ due to the low sample crystallinity and many peaks overlapping in the available *d* range. With the naked eye it is almost impossible to differentiate between single peaks (mirroring the proximity of the *a* and *b* lattice parameters) or to describe the background properly. Nonetheless, Figs. 9 ▸(*c*) and Fig. 9 ▸(*d*) provide evidence that the refined structural parameters describe the observed data very well. In particular, Fig. 9 ▸(*d*) makes it clear that the observed and calculated patterns match the difference pattern showing primarily noise. The seemingly higher noise in the difference pattern of Fig. 9 ▸(*d*) compared with that of Fig. 9 ▸(*e*) goes back to the smaller intensity range caused by differences in the normalization.

The corresponding *R*_Bragg_ values and parameters are also given in Table 1 ▸. More details of the spatial and isotropic displacement parameters are provided in Table 2 ▸.

## 6. Discussion

A closer look at the results of the diamond refinements from Table 1 ▸ yields that the one-dimensional and multi-dimensional refinements lead to very similar results. Although the cell parameters and volumes do not arrive at exactly the same values, their precisions are alike. The precision of the isotropic displacement parameters is alike as well. The lattice parameter published by Straumanis & Aka (1951 ▸), *a* = 3.5668 Å, also coincides with the multi-dimensional refinement result.

The results for the BaZn(NCN)_2_ sample point in the same direction: although the cell parameters and volumes in Table 1 ▸ are not quite identical, the precisions are alike, and this also relates to the precisions of the isotropic displacement parameters in Table 2 ▸. Additionally, the refined atomic positions for *all* atoms are identical within their tripled estimated standard deviations. The atomic displacement parameters are only larger than zero throughout in the multi-dimensional refinement.

The *R*_Bragg_ values given in Table 1 ▸ also indicate that those of the one-dimensional Rietveld refinement are smaller than those of the multi-dimensional refinement. This finding has already been noted and discussed (Jacobs *et al.*, 2015 ▸) and it may be a trivial consequence of the significantly larger number of data points used during the refinement in two dimensions. A similarly irritating size difference (but without physical meaning) between intensity-based *R* values (*wR*_2_) and structure-factor-based values (*R*_1_) was noted for single-crystal X-ray refinement when *SHELXL93* was released (Sheldrick, 2008 ▸).

Lastly, Table 1 ▸ highlights an interesting observation: the carbo­diimide phase crystallizing in *Pbca* is actually an intricate case for powder diffraction, simply due to the proximity of the *a* and *b* lattice parameters, differing by only 0.007 Å from powder XRD. Practically the same difference is found from the one-dimensional neutron refinement, 0.008 Å, mirroring the identical one-dimensional strategy. The two-dimensional neutron refinement, however, more clearly differentiates between *a* and *b*, by a five times larger 0.038 Å. One might think that this larger *a*/*b* difference goes back to the significantly higher number of data points and/or the better profile model which the two-dimensional approach can provide. In addition to that, a preliminary analysis of the internal structural coordinates shows that the two-dimensional refinement yields more balanced interatomic distances but slightly sharper angles in the carbo­diimide units. While the C—N bond lengths are scattered between 1.18 and 1.25 Å in the 1D case, the 2D approach gives 1.21–1.25 Å, with similar standard deviations of around 0.013 Å. Also, the two N—C—N units are less differently bent in the 1D results (173 and 175°) than in the 2D results (167 and 173°). With regard to the tetrahedral coordination of divalent Zn, it is less regular in the 1D results (1.99–2.06 Å) than in the 2D results (2.02–2.06 Å). Clearly, this needs a deeper investigation in the future.

These results demonstrate that multi-dimensional Rietveld refinement using a modified version of the *GSAS-II* software (and despite a partially defective detector) not only works but leads to results at least as precise as the conventional one-dimensional Rietveld refinement. This further proves that the reduction of raw measurement data as implemented in the *Mantid* software is working adequately.

## 7. Conclusion and outlook

In this study we have shown the results of the detector test for the new neutron TOF instrument POWTEX at FRM II, tested on the POWGEN instrument at SNS, Oak Ridge National Laboratory. The results also relate, indirectly, to other neutron TOF diffractometers with large-area detectors like the future DREAM beamline at the ESS.

After having overcome tremendous difficulties as a consequence of unfortunate transport damage to the detector, we were still able to measure several data sets including diamond, vanadium and BaZn(NCN)_2_.

A new data reduction routine for multi-dimensional data sets was implemented into the *Mantid* software and used, successfully, to reduce real-world multi-dimensional diffraction data using *Mantid* for the first time. These reduced multi-dimensional data sets were then refined, also for the first time, using a modified version of the *GSAS-II* software. The refinements of both diamond and BaZn(NCN)_2_ went smoothly, and their results were compared with the conventional one-dimensional approach for the same data sets. The precision of the refined parameters was similar in the one- and multi-dimensional refinements for both data sets. Additionally, the refined atomic positions of BaZn(NCN)_2_ were identical within three standard deviations, with subtle differences in their internal structural coordinates. Hence, there is clear evidence that the multi-dimensional Rietveld refinement yields at least the same precision as the one-dimensional Rietveld refinement.

In addition, the proof of concept for multi-dimensional data reduction using MATLAB has been transferred to publicly available (open source) data reduction code in the widely used *Mantid* software. Multi-dimensional Rietveld refinement was also transformed from the proof of concept utilizing MATLAB (Jacobs *et al.*, 2015 ▸) to a modified, not yet published, version of the widely used software suite *GSAS-II*. A more technical description of the modified version will be published in a separate article once the code is publicly released. While all basic refinement steps are functioning nicely within *GSAS-II*, we are currently working to extend the refinement options towards those examples for which more demanding effects must be treated. For example, the present BaZn(NCN)_2_ data set already posed a few challenges concerning the description of the multi-dimensional background, which was straightforwardly solved by providing the option of a manual background created from user-supplied base points. The reason for the more pronounced difference between the *a* and *b* lattice parameters in the two-dimensional refinement of BaZn(NCN)_2_ and its resulting internal coordinates compared with the conventional refinement also needs to be investigated carefully. Further sample effects like stress/strain and hydrogen background will be investigated in the future; the latter incorporates a peculiar 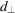 dependency which makes it uniquely treatable only within a multi-dimensional refinement.

Full data for the one- and two-dimensional refinements of diamond and BaZn(NCN)_2_ are provided in (extended) CIF format in the supporting information.

## Supplementary Material

ZIP archive of 6 powder CIF files. DOI: 10.1107/S1600576723002819/tu5033sup1.zip

## Figures and Tables

**Table 1:** Refinement results for the conventional one-dimensional (1D) and multi-dimensional (2D) refinements of diamond and BaZn(NCN)_2_ with corresponding *R*_Bragg_ values In brackets, three times the estimated standard deviation is shown for the last digit.

	Diamond, 1D	Diamond, 2D	BaZn(NCN)_2_, 1D	BaZn(NCN)_2_, 2D
*a* (Å)	3.57162 (8)	3.56663 (3)	11.953 (4)	12.025 (4)
*b* (Å)	3.57162 (8)	3.56663 (3)	11.945 (4)	11.987 (5)
*c* (Å)	3.57162 (8)	3.56663 (3)	6.855 (2)	6.871 (2)
*V* (Å^3^)	45.561 (4)	45.371 (1)	978.86 (4)	990.53 (4)
*R*_Bragg_	3.36%	7.49%	1.35%	7.74%
No. of points	2368	343 521	2368	21 943

**Table 2:** Atomic positions in fractional coordinates and isotropic displacement parameters for BaZn(NCN)_2_ The upper values correspond to the one-dimensional refinement and the lower values to the multi-dimensional refinement. In the brackets we provide tripled estimated standard deviations for the last digit.

	*x*	*y*	*z*	*U*_iso_ (Å^2^)
Ba	0.846 (3)	0.869 (3)	0.029 (4)	−0.001 (5)
	0.846 (2)	0.872 (2)	0.030 (4)	0.0010 (4)
Zn	0.920 (3)	0.122 (3)	0.283 (5)	0.006 (5)
	0.920 (3)	0.125 (4)	0.283 (5)	0.010 (4)
C1	0.368 (3)	0.163 (3)	0.601 (4)	0.005 (4)
	0.366 (2)	0.161 (3)	0.609 (4)	0.008 (4)
C2	0.613 (3)	0.424 (3)	0.522 (5)	0.006 (4)
	0.613 (3)	0.426 (3)	0.523 (5)	0.012 (4)
N1	0.653 (3)	0.494 (2)	0.416 (4)	0.017 (4)
	0.656 (3)	0.496 (2)	0.416 (4)	0.018 (4)
N2	0.419 (2)	0.095 (2)	0.512 (3)	0.011 (4)
	0.418 (2)	0.095 (2)	0.511 (3)	0.014 (4)
N3	0.317 (3)	0.243 (2)	0.678 (4)	0.015 (5)
	0.316 (3)	0.241 (2)	0.676 (4)	0.017 (5)
N4	0.579 (3)	0.348 (2)	0.627 (3)	0.013 (5)
	0.580 (2)	0.349 (2)	0.630 (3)	0.015 (4)
